# Impact of CT-Determined Sarcopenia and Body Composition on Survival Outcome in Patients with Advanced-Stage High-Grade Serous Ovarian Carcinoma

**DOI:** 10.3390/cancers12030559

**Published:** 2020-02-28

**Authors:** Se Ik Kim, Taek Min Kim, Maria Lee, Hee Seung Kim, Hyun Hoon Chung, Jeong Yeon Cho, Yong Sang Song

**Affiliations:** 1 Department of Obstetrics and Gynecology, Seoul National University College of Medicine, Seoul 03080, Korea; seikky@naver.com (S.I.K.); marialeemd@gmail.com (M.L.); bboddi0311@gmail.com (H.S.K.); chhkmj1@snu.ac.kr (H.H.C.); 2 Department of Radiology, Seoul National University College of Medicine, Seoul 03080, Korea; kjambong@gmail.com (T.M.K.); radjycho@snu.ac.kr (J.Y.C.); 3 Cancer Research Institute, Seoul National University College of Medicine, Seoul 03080, Korea

**Keywords:** ovarian neoplasms, high-grade serous carcinoma, sarcopenia, body composition, prognosis, survival

## Abstract

This study aimed to investigate the impact of sarcopenia and body composition on survival outcomes in Korean patients with advanced-stage high-grade serous ovarian carcinoma (HGSOC). We retrospectively identified patients diagnosed with and treated for International Federation of Gynecology and Obstetrics stage III-IV HGSOC. Skeletal muscle index (SMI) was measured using pre-treatment computed tomography scans at the third lumbar vertebra. Sarcopenia was defined as SMI <39.0 cm^2^/m^2^. Patients’ clinicopathologic characteristics and survival outcomes were compared according to sarcopenia presence. For subgroup analysis, we also measured the total fat area from the same image. In total, 76 and 103 patients were assigned to the sarcopenia and control groups, respectively. Comorbidities, stage, serum CA-125 levels, and size of residual tumor after surgery were similar between both groups. After a median follow up of 42.7 months, both groups showed similar progression-free survival (PFS) and overall survival (OS). In subgroup analysis confined to the sarcopenia group, patients with high fat-to-muscle ratio (FMR; ≥2.1, *n* = 38) showed significantly worse OS than those with low FMR (<2.1, *n* = 38) (5-year survival rate, 44.7% vs. 80.0%; *p* = 0.046), whereas PFS was not different (*p* = 0.365). Multivariate analyses identified high FMR as an independent poor prognostic factor for OS in this group (adjusted hazard ratio, 3.377; 95% confidence interval, 1.170–9.752; *p* = 0.024). In conclusion, sarcopenia did not influence recurrence rates and survival in Korean patients with advanced-stage HGSOC. However, among the patients with sarcopenia, high FMR was associated with decreased OS.

## 1. Introduction

Ovarian cancer, one of the deadliest gynecologic malignancies, causes more than 150,000 deaths worldwide each year [1]. The incidence of ovarian cancer is higher among high Human Development Index countries, and it is gradually increasing in Korea [2]. Owing to the absence of cancer-specific symptoms and effective screening tools, ovarian cancer tends to be diagnosed at an advanced-stage and thus has a high recurrence rate and poor five-year survival rate despite intensive treatment [3].

Sarcopenia, characterized by loss of skeletal muscle mass and function, does not occur exclusively in the elderly but is also commonly observed in cancer patients [4]. Previous studies have suggested sarcopenia as a prognostic factor associated with poor survival and increased resistance and toxicity to chemotherapy in patients with various malignancies, including breast, small cell lung, urothelial, and gastric cancers [5,6,7,8]. In ovarian cancer, conflicting results have been reported: while some studies concluded that sarcopenia adversely affected patients’ progression-free survival (PFS) or overall survival (OS) [9,10], others could not determine a significant association of sarcopenia with survival outcomes [11,12]. There were differences in study design, population, disease setting, and definition of sarcopenia among the studies; therefore, careful attention is required to interpret the study results. Moreover, considering the fact that body composition is different among the Organisation for Economic Co-operation and Development (OECD) member countries [13], sarcopenia and its impact on cancer prognosis may vary by geographical regions and ethnicities.

To determine sarcopenia, recent studies have suggested utilization of computed tomography (CT) scans. A cross-sectional image of CT scans at the level of the third lumbar vertebra (L3) is known to represent an individual’s body composition, such as total body skeletal muscle and adipose tissues and fat distribution [14,15]. Moreover, CT scans are acquired routinely as part of cancer patients’ care, so quantification of body composition using CT scans is quite possible.

To our knowledge, clinical significance of sarcopenia in Korean ovarian cancer patients has not been explored. Thus, we aimed to investigate impact of sarcopenia on survival outcomes in Korean patients with advanced-stage high-grade serous ovarian carcinoma (HGSOC), which is the predominant histologic type of ovarian cancer. In this study, sarcopenia was determined based on the pre-treatment CT scan; the fat composition was also measured considering the fact that the Asian population generally has a higher body fat percentage than the Western population at the same body mass index (BMI) [16].

## 2. Results

### 2.1. Analysis in All Patients

Patients’ clinicopathologic characteristics are presented in Table 1. The sarcopenia group (*n* = 76) had significantly lower pre-treatment body mass index (BMI) (mean, 22.1 vs. 24.7 kg/m^2^; *p* < 0.001) and received neoadjuvant chemotherapy (NAC) less frequently (17.1% vs. 30.1%; *p* = 0.046), compared to the control group (*n* = 103). Other characteristics showed no significant difference between two groups. The patients’ initial body composition and laboratory results are presented in Table 2. The sarcopenia group showed less skeletal muscle area (median, 88.1 vs. 106.1 cm^2^; *p* < 0.001) and total fat area (median, 188.5 vs. 230.7 cm^2^; *p* < 0.001). Among the various calculated body composition indices, all others except skeletal muscle index (SMI) were similar between the sarcopenia and control groups. There were no differences in the laboratory results, inflammatory indices, and nutritional index between the groups. 

**Figure 1:**
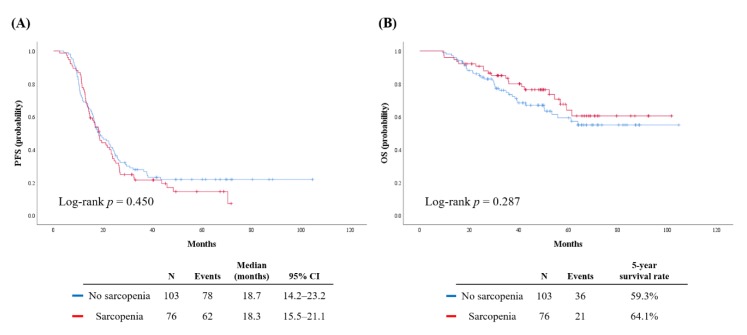
Survival outcomes of patients. (**A**) Progression-free survival; (**B**) overall survival.

The median length of observation was 42.7 months, and it was not different between both groups (45.9 vs. 41.5 months; *p* = 0.497). During this period, 140 patients (78.2%) experienced disease recurrence, and 57 patients (31.8%) died of disease. Patients in the sarcopenia and control groups showed similar PFS (median, 18.3 vs. 18.7 months; *p* = 0.450; Figure 1A) and OS (five-year survival rate, 64.1% vs. 59.3%; *p* = 0.287; Figure 1B). 

Multivariate analyses adjusting patients’ age, International Federation of Gynecology and Obstetrics (FIGO) stage, serum CA-125 levels, primary treatment strategy, residual tumor size after surgery, and BMI revealed that pre-treatment sarcopenia status did not influence patients’ PFS and OS (Table 3). Instead, age ≥58 years (adjusted hazard ratio (aHR), 1.458; 95% confidence interval (CI), 1.024–2.077; *p* = 0.037) and gross residual tumor (aHR, 1.504; 95% CI, 1.068–2.119; *p* = 0.020) were identified as independent poor prognostic factors for PFS. For OS, NAC rather than primary debulking surgery (PDS; aHR, 2.000; 95% CI, 1.096–3.649; *p* = 0.024) and gross residual tumor (aHR, 2.142; 95% CI, 1.258–3.647; *p* = 0.005) were the poor prognostic factors.

### 2.2. Subgroup Analysis in Sarcopenia Patients

**Figure 2:**
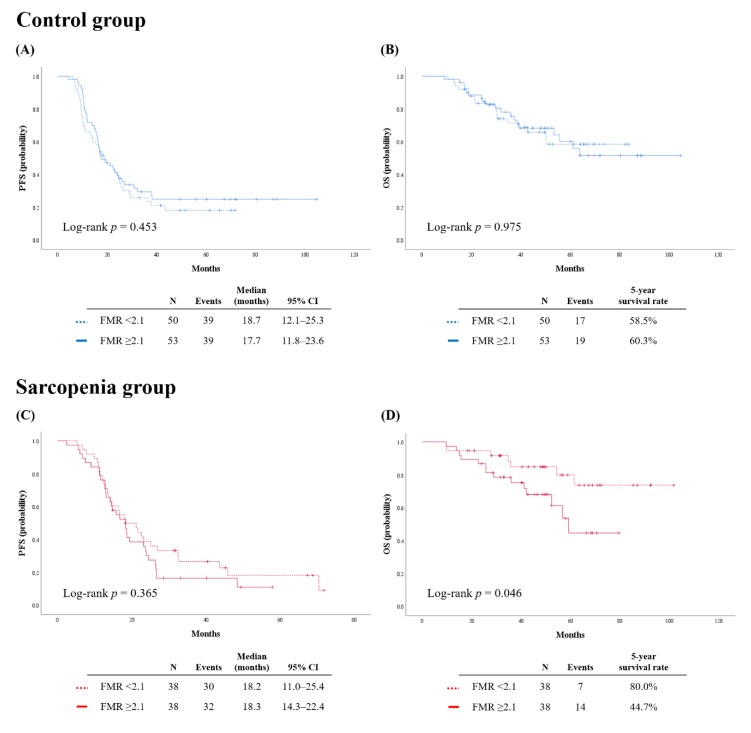
Survival outcomes of patients by fat-to-muscle ratio. (Upper) Control group; (Lower) sarcopenia group. (**A**,**C**) Progression-free survival; (**B**,**D**) overall survival.

Because we focused on patients’ relative fat mass, we adopted fat-to-muscle ratio (FMR) among the calculated body composition indices. As the median FMR of all patients was 2.1, we subdivided the patients into FMR low (<2.1) and high (≥2.1) groups. Among the patients without sarcopenia (*n* = 103), no differences in PFS and OS were observed between the FMR low and high groups (*p* = 0.453 and *p* = 0.975, respectively) (Figure 2A,B).

Next, we performed subgroup analysis confined to the sarcopenia group (*n* = 76). Patients’ clinicopathologic characteristics are presented in Table 4. Compared to patients with low FMR, patients with high FMR were significantly older (mean, 60.1 vs. 54.0 years; *p* = 0.006), and had higher pre-treatment BMI (mean, 23.6 vs. 20.7 kg/m^2^; *p* < 0.001) and prevalence of dyslipidemia (15.8% vs. 0%; *p* = 0.025). Other characteristics were similar between the FMR high and low groups. Sarcopenia patients’ initial body composition and laboratory results are presented in Table 5. Compared to the FMR low group, the FMR high group showed higher total fat area (median, 228.1 vs. 141.5 cm^2^; *p* < 0.001) and visceral-to-subcutaneous fat ratio (VSR; median, 0.6 vs. 0.3; *p* = 0.001), and lower skeletal muscle mass-to-visceral fat ratio (SVR; median, 1.1 vs. 2.5; *p* < 0.001). However, skeletal muscle area as well as SMI were similar between both groups. There were no differences in the laboratory results, inflammatory indices, and nutritional index between the two groups.

In the sarcopenia group, patients with FMR showed significantly worse OS than those with low FMR (five-year survival rate, 44.7% vs. 80.0%; *p* = 0.046), whereas PFS was not different (*p* = 0.365) (Figure 2C,D). Multivariate analyses identified high FMR as an independent poor prognostic factor for OS in this group (aHR, 3.377; 95% CI, 1.170–9.752; *p* = 0.024), whereas high FMR did not influence patients’ PFS (*p* = 0.825) (Table 6). Other poor prognostic factors for OS were NAC rather than PDS (aHR, 3.310; 95% CI, 1.096–10.000; *p* = 0.034) and gross residual tumor after surgery (aHR, 4.377; 95% CI, 1.655–11.578; *p* = 0.003).

### 2.3. Correlations between Body Compositio and Systemic Inflammatory Indices

**Figure 3:**
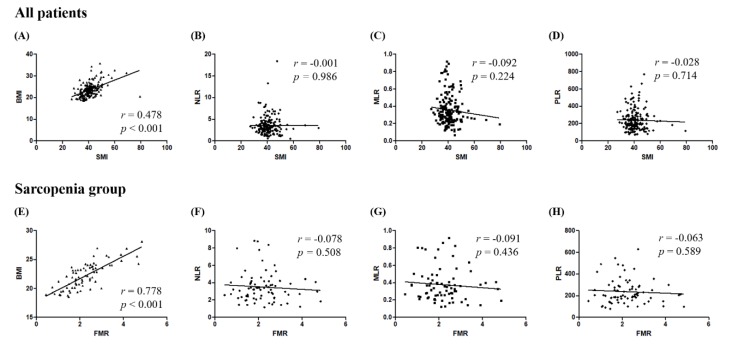
Correlations between body composition and systemic inflammatory indices. (Upper) Analyses according to skeletal muscle index in all patients; (Lower) analyses according to fat to muscle ratio in sarcopenia patients. (**A**,**E**) Body mass index; (**B**,**F**) neutrophil-to-lymphocyte ratio; (**C**,**G**) monocyte-to-lymphocyte ratio; (**D**,**H**) platelet-to-lymphocyte ratio.

We investigated the correlations between SMI and the three systemic inflammatory indices, neutrophil-to-lymphocyte ratio (NLR), monocyte-to-lymphocyte ratio (MLR), and platelet-to-lymphocyte ratio (PLR). While SMI was significantly associated with BMI (Pearson’s correlation coefficient *r* = 0.478; *p* < 0.001), there were no correlations between SMI and either NLR, MLR, or PLR (Figure 3A–D).

To elucidate the underlying mechanisms of high FMR and poor survival outcome in sarcopenia patients, correlations between FMR and the three systemic inflammatory indices—NLR, MLR, and PLR—were also investigated. While FMR was significantly associated with BMI (Pearson’s correlation coefficient *r* = 0.778; *p* < 0.001), significant correlations were not observed between FMR and NLR, between FMR and MLR, and between FMR and PLR (Figure 3E–H).

## 3. Discussion

In this study, we investigated the impact of pre-treatment sarcopenia on survival outcomes in patients with advanced-stage HGSOC and revealed that there was no significant association between sarcopenia and recurrence rate or survival. However, further subgroup analysis identified high FMR as a poor prognostic factor for OS in sarcopenia patients.

Unlike other malignancies in which sarcopenia is associated with decreased OS and increased post-operative morbidity [17,18], inconsistent results on the relationship between sarcopenia and survival outcome are observed among the studies regarding ovarian cancer. There are two representative retrospective studies: while Bronger et al. reported the baseline sarcopenia is an independent poor prognostic factor for PFS and OS in advanced-stage serous ovarian cancer [10], Rutten et al. demonstrated that sarcopenia was not a prognostic factor for OS or major complications in ovarian cancer patients undergoing PDS [11]. Most studies were conducted in Western populations whose body composition is different from that of Asians. Recently, a Japanese retrospective study showed results similar to those of our study; pre-treatment SMI was not associated with ovarian cancer patients’ PFS and OS [19]. However, that study included early-stage disease and histologic types other than HGSOC, which is definitely different compared to our study.

To date, researches on sarcopenia in cancer patients have been conducted in the context of cancer cachexia. Patients with HGSOC are at high risk of sarcopenia and cachexia. First, as the disease is often detected in a much-progressed state, the patients might already have cachexia at the time of diagnosis. Second, an enlarging tumor mass induces metabolic dysfunction towards catabolism, while bowel obstructions during disease progression cause anorexia or reduced food intake [20]. Third, newly diagnosed patients undergo aggressive cytoreductive surgery followed by taxane- and platinum-based chemotherapy as an established standard of care, which further aggravate anorexia and loss of body weight [21]. Consequently, poor nutritional status and loss of muscle mass and strength is highly expected in patients with ovarian cancer. Previously, our research team reported that underweight status, one of the representative features of cachexia, was a poor prognostic factor in patients with advanced-stage ovarian cancer [22]. In the current study, rather than cancer cachexia, we focused on sarcopenia itself which may be incidentally discovered at the time of diagnosis of ovarian cancer. For this purpose, we excluded pre-treatment underweight patients in whom cancer cachexia could already be dominant.

CT scans are known to distinguish fat and muscle tissue accurately with high reproducibility by using specific attenuation of each tissue [23]. The most commonly used and validated Hounsfield unit (HU) range for adipose tissue is −190 to −30. However, there is an inconsistency between the literature with the HU range for muscle tissue, which starts from either 0 or −29 and ends at 100 or 150. Exclusion of the area ranging from −29 to 0 HU may result in significant loss of the total muscle area. Instead, we used −29 to 150 HU for muscle tissue so as not to miss the low attenuation muscle, same as that used in previous studies [24,25].

Although there was no statistical difference in PFS and OS between the sarcopenia and control groups, we found that high FMR is an independent prognostic factor for OS in the sarcopenia group. The coexistence of sarcopenia and obesity (sarcopenic obesity) seems to affect patients’ survival outcomes equal to or greater than the sum of the respective risks of obesity and sarcopenia alone [26]. A previous study has reported that the presence of sarcopenic obesity increased patients’ mortality in colorectal cancer [27]. In the current study, we focused on amount of the fat relative to the muscle, rather than BMI, considering the fact that Asians have a higher body fat percentage than Westerners at the same BMI [16], and similar results were found with the previous studies.

One remarkable observation in the current study is that we tried to elucidate the mechanisms underlying the relationship between high FMR and decreased survival in sarcopenic patients with advanced-stage HGSOC. Previously, our research team reported that adipose stem cells from visceral and subcutaneous fat facilitated the growth and migration of ovarian cancer cells via IL-6/JAK2/STAT3 pathway [28]. Adding to this, other researchers have reported that visceral obesity is associated with a chronic inflammatory state, which leads to adverse metabolic consequences [29]. The relationship between sarcopenia and systemic inflammation has been also reported [30]. In this context, we hypothesized that systemic inflammatory indices (NLR, MLR, and PLR) would be different between the high and low FMR groups. However, there were no differences between both groups, and correlations were not observed between FMR and the three systemic inflammatory indices. Similar correlations were also observed between SMI and the inflammatory indices. These findings might be related to the small sample size or exclusion of underweight patients. Moreover, investigation of other systemic inflammatory markers and adipose tissue-derived cytokines, such as leptin, IL-6 and TNF-α, may answer our hypothesis exactly. 

In keeping with the era of precision medicine, early identification of adverse body composition which might influence patients’ survival outcome would be one of the important issues. For patients who have high FMR, aerobic exercises may be recommended to reduce adipose tissue. To date, intervention studies to prevent sarcopenia or maintain skeletal muscle mass in patients with ovarian cancer is still insufficient. Nevertheless, as recommended by various societies, prescription of resistance-type exercise training and a protein-rich diet or protein supplement should be also considered for HGSOC patients with sarcopenia. Hormone replacement therapy or vitamin D may be given, but more evidence is needed [31,32,33]. For those who have chemotherapy-induced nausea and vomiting, adequate anti-emetics as well as parenteral nutrition should be provided. If patients suffer from dyspepsia or abdominal distention owing to large amount of ascites, drainage of ascitic fluid may improve patients’ symptoms as well as nutritional status. If there is long persistent seeding ileus, procedures such as stoma formation may be considered as well. Prior to administering these interventions, all HGSOC patients should be screened for sarcopenia and adiposity at the time of diagnosis. As pre-treatment or baseline CT scans are commonly performed to determine the severity of disease and to establish a treatment plan in most patients, routine screening for body composition would be available and practical.

The current study has several limitations. First, a small sample size with possible selection bias that originates from the retrospective study design might be problematic. Second, the sequential change of body composition in each individual was not considered. Third, associations between sarcopenia and surgery or chemotherapy-related complications were not investigated. Finally, although muscle mass was successfully measured by using CT scans, muscle quality was hard to know by this imaging modality. Decreased muscle quality is known to be associated with the fatty degeneration or fatty infiltration of the muscle (i.e., myosteatosis). Currently, magnetic resonance imaging (MRI) is the best modality to evaluate the muscle quality and myosteatosis. In addition, MRI may also provide information on inflammation, edema, fibrosis, and atrophy in the muscle [34,35,36]. However, because of its high cost, limited availability, and long image acquisition time, MRI-based body composition assessment is not a routine clinical practice. Most of our study population did not undergo pre-treatment MRI, so accurate assessment of muscle quality was unavailable. Despite this study’s limitations, the current study is the first study to adopt CT-based body composition measurement techniques to identify prognostic factors in Korean ovarian cancer patients.

## 4. Materials and Methods 

This retrospective cohort study was approved by the Institutional Review Board of Seoul National University Hospital (SNUH; No. H-1911-171-1082) which waived the requirement to obtain informed consent.

### 4.1. Study Population

From the Ovarian Cancer Cohort Database, we searched patients who met the following inclusion criteria: (1) patients older than 18 years of age, (2) those with HGSOC diagnosed and primarily treated at SNUH between January 2010 and December 2017, and (3) those with FIGO stage III-IV disease. However, patients with the following conditions were excluded: (1) patients with any malignancy other than HGSOC, (2) those with insufficient clinical data, (3) those who did not undergo pre-treatment CT scans, and (4) those who were underweight based on pre-treatment BMI (<18.5 kg/m^2^). In total, 179 patients who met these criteria were included in this analysis.

### 4.2. CT Image Analysis and Definition of Sarcopenia

**Figure 4:**
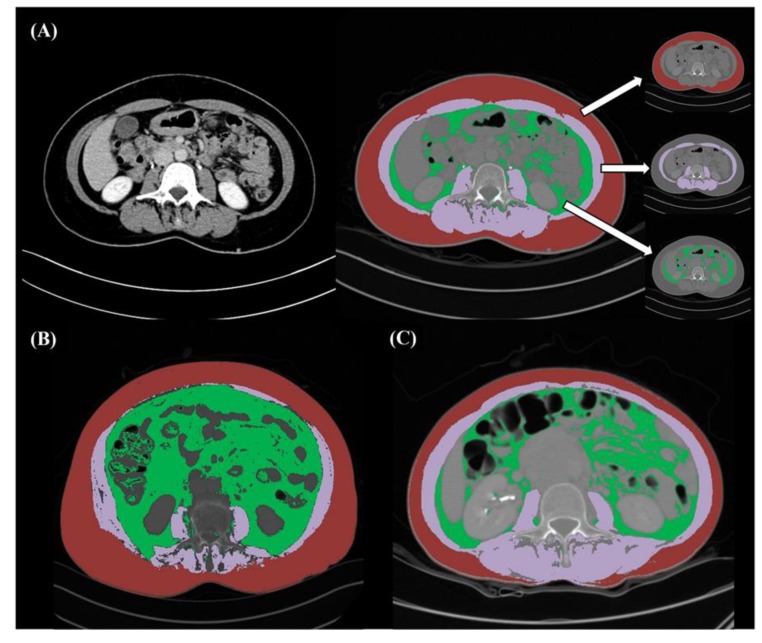
Evaluation of body composition using CT image. Preoperative axial CT image at the level of L3 vertebral body level. (**A**) A 52-year old woman with newly diagnosed high-grade serous ovarian carcinoma. Total abdominal muscle area (purple), visceral fat area (green), and subcutaneous fat area (red) are segmented by the semi-automatic technique; (**B**) A 73-year old woman with sarcopenia and high fat-to-muscle ratio (4.6); (**C**) A 53-year old woman with sarcopenia and low fat-to-muscle ratio (1.5).

For the evaluation of sarcopenia, a cross-sectional area of the muscle at the level of L3 vertebral body was measured using baseline CT scans. Applying previously validated boundaries of −190 to −30 HU for fat tissue and −29 to 150 HU for skeletal muscle [37], an experienced radiologist (T.M.K., 5 years of genitourinary imaging experience) who was blinded to the clinical outcome measured total abdominal muscle area (cm^2^), intramuscular fat area (cm^2^), visceral fat area (cm^2^), and subcutaneous fat area (cm^2^). This CT image analysis was conducted by semi-automatic technique using AsanJ-Morphometry software (Asan Image Metrics, Seoul, Korea) (Figure 4A–C).

Total abdominal muscle area (cm^2^) was normalized for height (m^2^) and reported as lumbar SMI. To date, the sex-specific cut-off values of SMI for sarcopenia have not been validated in Korean healthy individuals. Adoption of the cut-off values suggested by Japanese study groups was deterred because they were developed in different study populations (e.g., patients with liver disease, [38]) or had age limitations (e.g., <50 years, [39]). In addition, proportions of populations with overweight-obesity are even different between Korea and Japan according to the OECD Health Statistics 2019 [13]. Therefore, we defined sarcopenia as SMI of <39.0 cm^2^/m^2^ according to the proposed cut-off value by an international consensus, and divided patients into sarcopenia group (<39.0 cm^2^/m^2^) and no sarcopenia group (control group; ≥39.0 cm^2^/m^2^) [40]. We also calculated other body composition indices, such as FMR, VSR, and SVR.

### 4.3. Data Collection

We collected patients’ clinicopathologic characteristics including age, co-morbidities such as hypertension or diabetes, American Society of Anesthesiologists score, FIGO stage, NAC, residual tumor size after PDS or interval debulking surgery, and regimens and cycles of adjuvant chemotherapy. Patients treated with neoadjuvant chemotherapy received 3–4 cycles of taxane- and platinum-based chemotherapy before surgery, and optimal debulking surgery was considered when no gross residual tumor was achieved. 

Patients’ pre-treatment BMI was calculated as body weight (kg) divided by height squared (m^2^), which were measured at the time of diagnosis. All patients were classified into three groups based on the following BMI criteria suggested by the World Health Organization for the Asian population: normal (≥18.5 kg/m^2^ and <23.0 kg/m^2^), overweight (≥23.0 kg/m^2^ and <25.0 kg/m^2^), and obese (≥25.0 kg/m^2^) [16].

Data acquisition also included serum CA-125 levels, hemoglobin, albumin, and differential blood cell counts including neutrophils, lymphocytes, monocytes, and platelets at initial diagnosis, less than a month prior to either PDS or the start date of NAC. As systemic inflammatory indices, we calculated the NLR, MLR, and PLR. As a pre-treatment nutritional index, we calculated the prognostic nutritional index (PNI) as follows: 10 × serum albumin (g/dL) + 0.005 × peripheral blood lymphocyte count (/uL) [41].

In terms of survival data, OS was defined as the time interval between the date of diagnosis and the date of cancer-related death or the end of the study. During the surveillance, patients received CT scanning routinely every three to four months for the first two years, every six months for the next two years, and thereafter, every year or when symptoms or examination findings were suspicious for recurrence. Therefore, we defined PFS as the time interval between the start date of primary treatment and the date of image-confirmed disease progression, which was assessed based on the Response Evaluation Criteria in Solid Tumors (RECIST) version 1.1 [42].

### 4.4. Statistical Analysis

We compared the patients’ clinicopathologic characteristics and survival outcomes between the sarcopenia and control groups. We used Student’s t-test and the Mann-Whitney U test for comparisons of continuous variables and Pearson’s chi-squared and Fisher’s exact test for categorical variables. For survival analysis, we conducted the Kaplan-Meier methods with log-rank test. Multivariate analysis was performed using a Cox proportional-hazards model, and aHRs and 95% CIs were calculated. We used IBM SPSS Statistics software (version 25·0; SPSS Inc., Chicago, IL, USA) for these analyses. Correlation values were calculated by the Pearson’s correlation coefficient test using the GraphPad Prism 5 software (GraphPad Inc., La Jolla, CA, USA). A *p* value <0.05 was considered statistically significant.

## 5. Conclusions

In conclusion, we investigated the clinical significance of sarcopenia in Korean patients with advanced-stage HGSOC and found that sarcopenia did not influence patients’ recurrence rates and survival. However, among the sarcopenia patients, those who had relatively high levels of fat compared to muscle mass showed worse OS. Further translational researches and prospective studies are warranted.

## Figures and Tables

**Table 1:** Clinicopathologic characteristics of all patients.

Characteristics	All (*n* = 179, %)	No Sarcopenia Group (*n* = 103, %)	Sarcopenia Group (*n* = 76, %)	*p*
Age, years				
Mean ± SD	57.5 ± 10.6	57.8 ± 11.1	57.0 ± 9.9	0.615
BMI ^1^, kg/m^2^				
Mean ± SD	23.6 ± 3.2	24.7 ± 3·3	22.1 ± 2.3	<0.001
Normal (18.5−22.9)	81 (45.3)	35 (34.0)	46 (60.5)	<0.001
Overweight (23.0–24.9)	52 (29.1)	30 (29.1)	22 (28.9)	
Obesity (≥25.0)	46 (25.7)	38 (36.9)	8 (10.5)	
Comorbidities				
Hypertension	48 (26.8)	28 (27.2)	20 (26.3)	0.897
Diabetes	15 (8.4)	10 (9.7)	5 (6.6)	0.455
Dyslipidemia	21 (11.7)	15 (14.6)	6 (7.9)	0.171
ASA score				0.080
1	63 (35.2)	31 (30.1)	32 (42.1)	
2	104 (58.1)	67 (65.0)	37 (48.7)	
3	12 (6.7)	5 (4.9)	7 (9.2)	
FIGO stage				0.653
IIIA1	8 (4.5)	5 (4.9)	3 (3.9)	
IIIA2	6 (3.4)	4 (3.9)	2 (2.6)	
IIIB	17 (9.5)	9 (8.7)	8 (10.5)	
IIIC	91 (50.8)	50 (48.5)	41 (53.9)	
IVA	10 (5.6)	4 (3.9)	6 (7.9)	
IVB	47 (26.3)	31 (30.1)	16 (21.1)	
CA-125, IU/ml				
Median (range)	801.0 (5.1–24720.0)	833.0 (7.0–10000.0)	793.0 (5.1–24720.0)	0.829
Primary treatment strategy				0.046
PDS	135 (75.4)	72 (69.9)	63 (82.9)	
NAC	44 (24.6)	31 (30.1)	13 (17.1)	
Residual tumour after PDS/IDS				0.336
No gross	114 (63.7)	67 (65.0)	47 (61.8)	
<1 cm	44 (24.6)	26 (25.2)	18 (23.7)	
1–2 cm	10 (5.6)	3 (2.9)	7 (9.2)	
≥2 cm	11 (6.1)	7 (6.8)	4 (5.3)	
Regimen of first-line chemotherapy				0.368
Paclitaxel-Carboplatin	161 (89.9)	93 (90.3)	68 (89.5)	0.393
Docetaxel-Carboplatin	14 (7.8)	9 (8.7)	5 (6.6)	
Paclitaxel-Carboplatin-Bevacizumab	4 (2.2)	1 (1.0)	3 (3.9)	
Main cycles of first-line chemotherapy				
Median (range)	6 (4–12)	6 (4–12)	6 (4–12)	0.438
4–6	123 (68.7)	70 (68.0)	53 (69.7)	
7–9	50 (27.9)	31 (30.1)	19 (25.0)	
10–12	6 (3.4)	2 (1.9)	4 (5.3)	
Recurrence	140 (78.2)	78 (75.7)	62 (81.6)	0.349
PSR ^2^	95 (53.1)	47 (45.6)	48 (63.2)	0.031
PRR	45 (25.1)	31 (30.1)	14 (18.4)	
Platinum sensitivity				0.075
Platinum-sensitive ^3^	134 (74.9)	72 (69.9)	62 (81.6)	
Platinum-resistant	45 (25.1)	31 (30.1)	14 (18.4)	

^1^ In this study, underweight patients (BMI <18.5 kg/m^2^) were excluded in analysis. ^2^ PSR was defined as relapse ≥6 months after completion of taxane- and platinum-based chemotherapy, whereas PRR as relapse <6 months.^3^ In addition to PSR, the patients who completed taxane- and platinum-based chemotherapy and did not experience disease recurrence during at least six months of follow-up period were considered platinum-sensitive. Abbreviations: ASA, American Society of Anesthesiologists; BMI, body mass index; CA-125, cancer antigen 125; FIGO, International Federation of Gynecology and Obstetrics; IDS, interval debulking surgery; NAC, neoadjuvant chemotherapy; PDS, primary debulking surgery; PRR, platinum-resistant recurrence; PSR, platinum-sensitive recurrence; SD, standard deviation.

**Table 2:** Body composition and laboratory results of all patients.

Characteristics	All (*n* = 179, %)	No Sarcopenia Group (*n* = 103, %)	Sarcopenia Group (*n* = 76, %)	*p*
Body composition at diagnosis ^1^				
Skeletal muscle area, cm^2^	98.0 (64.1–209.8)	106.1 (84.8–209.8)	88.1 (64.1–109.0)	<0.001
Total fat area, cm^2^	211.8 (42.2–612.5)	230.7 (78.8–612.5)	188.5 (42.2–458.2)	<0.001
Subcutaneous fat	131.7 (34.4–310.8)	154.0 (55.8–310.8)	119.8 (34.4–252.0)	<0.001
Visceral fat	70.4 (6.6–289.4)	81.5 (11.2–289.4)	59.6 (6.6–213.0)	0.001
Muscle fat	6.2 (0.7–36.2)	6.5 (0.7–36.2)	5.3 (1.2–31.6)	0.103
Calculated body composition index ^1^				
Skeletal muscle index (SMI), cm^2^/m^2^	40.3 (27.1–79.2)	42.6 (39.0–79.2)	36.3 (27.1–39.0)	<0.001
Fat-to-muscle ratio (FMR)	2.1 (0.5–6.5)	2.1 (0.8–6.5)	2.1 (0.5–4.9)	0.508
Visceral-to-subcutaneous fat ratio (VSR)	0.5 (0.1–2.9)	0.5 (0.1–1.4)	0.4 (0.1–2.9)	0.212
Skeletal muscle mass-to-visceral fat ratio (SVR)	1.4 (0.3–14.2)	1.3 (0.3–8.7)	1.5 (0.5–14.2)	0.178
Laboratory test at diagnosis ^1^				
Hemoglobin, g/dL	12.2 (8.3–14.9)	12.2 (9.1–14.9)	12.4 (8.3–14.6)	0.491
WBC count, 10^3^/uL	7.0 (1.5–17.0)	6.9 (1.5–14.7)	7.1 (3.5–17.0)	0.417
Neutrophil (%)	68.9 (28.0–92.0)	68.9 (28.0–92.0)	68.9 (47.0–83.0)	0.734
Lymphocyte (%)	21.7 (5.0–57.0)	22.2 (5.0–57.0)	21.2 (9.4–42.9)	0.772
Monocyte (%)	6.8 (0.7–20.9)	6.8 (0.7–20.9)	6.9 (3.7–16.0)	0.335
Platelet count, 10^3^/uL	316.5 (95.0–698.0)	312.0 (95.0–698.0)	323.0 (159.0–634.0)	0.355
Albumin, g/dL	3.9 (2.3–5.1)	3.8 (2.3–4.6)	4.0 (2.4–5.1)	0.128
Calculated inflammatory index ^1^				
Neutrophil-to-lymphocyte ratio (NLR)	3.2 (0.5–18.4)	3.1 (0.5–18.4)	3.2 (1.2–8.8)	0.945
Monocyte-to-lymphocyte ratio (MLR)	0.3 (0.1–0.9)	0.3 (0.1–0.9)	0.3 (0.1–0.9)	0.378
Platelet-to-lymphocyte ratio (PLR)	204.9 (71.6–768.5)	208.3 (71.6–768.5)	204.7 (77.2–628.1)	0.923
Calculated nutritional index				
Prognostic nutritional index (PNI) ^2^				
Mean ± SD	46.0 ± 7.0	45.3 ± 7.0	47.0 ± 6.9	0.100

^1^ Median (range). ^2^ PNI, 10 × serum albumin (g/dL) + 0.005 × peripheral blood lymphocyte count (/uL). Abbreviations: SD, standard deviation; WBC, white blood cell.

**Table 3:** Factors associated with patients’ survival outcomes.

Characteristics	*n*	(A) Progression-Free Survival						(B) Overall Survival					
		Univariate Analysis			Multivariate Analysis			Univariate Analysis			Multivariate Analysis		
		HR	95% CI	*p*	aHR	95% CI	*p*	HR	95% CI	*p*	aHR	95% CI	*p*
Age, years													
<58	94	1	−	−	1	−	−	1	−	−	1	−	−
≥58	85	1.558	1.116–2.175	0.009	1.458	1.024–2.077	0.037	1.551	0.919–2.618	0.101	1.213	0.692–2.127	0.500
FIGO stage													
III	122	1	−	−	1	−	−	1	−	−	1	−	−
IV	57	1.342	0.944–1.908	0.101	1.216	0.820–1.805	0.330	1.490	0.861–2.579	0.154	1.256	0.690–2.288	0.456
CA-125, IU/ml													
<800	89	1	−	−	1	−	−	1	−	−	1	−	−
≥800	90	1.164	0.835–1.622	0.370	1.140	0.811–1.602	0.451	1.110	0.660–1.867	0.695	0.964	0.560–1.660	0.894
Primary treatment strategy													
PDS	135	1	−	−	1	−	−	1	−	−	1	−	−
NAC	44	1.669	1.151–2.419	0.007	1.380	0.902–2.113	0.138	2.376	1.392–4.057	0.002	2.000	1.096–3.649	0.024
Residual tumor after PDS/IDS													
No gross	114	1	−	−	1	−	−	1	−	−	1	−	−
Gross	65	1.568	1.119–2.198	0.009	1.504	1.068–2.119	0.020	2.169	1.286–3.658	0.004	2.142	1.258–3.647	0.005
BMI, kg/m^2^													
Normal (18.5−22.9)	81	1	−	−	1	−	−	1	−	−	1	−	−
Overweight (23.0−24.9)	52	0679	0.449–1.029	0.068	0.656	0.429–1.004	0.052	0.728	0.366–1.450	0.367	0.707	0.347–1.437	0.338
Obesity (≥25.0)	46	1.184	0.799–1.755	0.399	1.132	0.742–1.726	0.564	1.638	0.909–2.951	0.100	1.261	0.661–2.405	0.481
Sarcopenia													
No	103	1	−	−	1	−	−	1	−	−	1	−	−
Yes	76	0.879	0629–1.228	0.451	1.292	0.906–1.843	0.157	0.747	0.436–1.280	0.289	0.870	0.488–1.550	0.636

Abbreviations: aHR, adjusted hazard ratio; BMI, body mass index; CA-125, cancer antigen 125; CI, confidence interval; FIGO, International Federation of Gynecology and Obstetrics; HR, hazard ratio; IDS, interval debulking surgery; NAC, neoadjuvant chemotherapy; PDS, primary debulking surgery.

**Table 4:** Clinicopathologic characteristics of sarcopenia patients. ^1^ In this study, underweight patients (BMI < 18.5 kg/m^2^) were excluded in analysis. ^2^ PSR was defined as relapse ≥6 months after completion of taxane- and platinum-based chemotherapy, whereas PRR as relapse <6 months. ^3^ In addition to PSR, the patients who completed taxane- and platinum-based chemotherapy and did not experience disease recurrence during at least six months of follow-up period were considered platinum-sensitive.

Characteristics	FMR Low Group (*n* = 38, %)	FMR High Group (*n* = 38, %)	*p*
Age, years			
Mean ± SD	54.0 ± 8.2	60.1 ± 10.6	0.006
BMI ^1^, kg/m^2^			
Mean ± SD	20.7 ± 1.5	23.6 ± 1.9	<0.001
Normal (18.5–22.9)	33 (86.8)	13 (34.2)	<0.001
Overweight (23.0–24.9)	5 (13.2)	17 (44.7)	
Obesity (≥25.0)	0	8 (21.1)	
Comorbidities			
Hypertension	7 (18.4)	13 (34.2)	0.118
Diabetes	3 (7.9)	2 (5.3)	>0.999
Dyslipidemia	0	6 (15.8)	0.025
ASA score			0.466
1	16 (42.1)	16 (42.1)	
2	20 (52.6)	17 (44.7)	
3	2 (5.3)	5 (13.2)	
FIGO stage			0.613
III	28 (73.7)	26 (68.4)	
IV	10 (26.3)	12 (31.6)	
CA-125, IU/ml			
Median (range)	793.0 (13.0–24720.0)	712.5 (5.1–7821.0)	0.949
Primary treatment strategy			0.361
PDS	33 (86.8)	30 (78.9)	
NAC	5 (13.2)	8 (21.1)	
Residual tumour after PDS/IDS			0.533
No gross	23 (60.5)	24 (63.2)	
<1 cm	11 (28.9)	7 (18.4)	
1–2 cm	2 (5.3)	5 (13.2)	
≥2 cm	2 (5.3)	2 (5.3)	
Regimen of first-line chemotherapy			0.306
Paclitaxel-Carboplatin	36 (94.7)	32 (84.2)	
Docetaxel-Carboplatin	1 (2.6)	4 (10.5)	
Paclitaxel-Carboplatin-Bevacizumab	1 (2.6)	2 (5.3)	
Main cycles of first-line chemotherapy			
Median (range)	6 (4–12)	6 (4–12)	0.374
4–6	28 (73.7)	25 (65.8)	0.725
7–9	8 (21.1)	11 (28.9)	
10–12	2 (5.3)	2 (5.3)	
Recurrence	30 (78.9)	32 (842)	0.554
PSR ^2^	24 (63.2)	24 (63.2)	0.638
PRR	6 (15.8)	8 (21.1)	
Platinum sensitivity			0.554
Platinum-sensitive ^3^	32 (84.2)	30 (78.9)	
Platinum-resistant	6 (15.8)	8 (21.1)	

Abbreviations: ASA, American Society of Anesthesiologists; BMI, body mass index; CA-125, cancer antigen 125; FIGO, International Federation of Gynecology and Obstetrics; IDS, interval debulking surgery; NAC, neoadjuvant chemotherapy; PDS, primary debulking surgery; PRR, platinum-resistant recurrence; PSR, platinum-sensitive recurrence; SD, standard deviation.

**Table 5:** Body composition and laboratory results of sarcopenia patients.^1^ Median (range). ^2^ PNI, 10 × serum albumin (g/dL) + 0.005 × peripheral blood lymphocyte count (/uL).

Characteristics	FMR low group (*n* = 38, %)	FMR high group (*n* = 38, %)	*p*
Body composition at diagnosis ^1^			
Skeletal muscle area, cm^2^	89.8 (74.5–109.0)	86.4 (64.1–104.8)	0.094
Total fat area, cm^2^	141.5 (42.2–199.3)	228.1 (166.0–458.2)	<0.001
Subcutaneous fat	97.1 (34.4–165.4)	138.3 (54.6–252.0)	<0.001
Visceral fat	35.2 (6.6–79.4)	82.1 (30.1–213.0)	<0.001
Muscle fat	3.8 (1.2–15.2)	7.8 (2.3–31.6)	<0.001
Calculated body composition index ^1^			
Skeletal muscle index (SMI), cm^2^/m^2^	36.0 (27.1–39.0)	37.4 (28.7–39.0)	0.228
Fat-to-muscle ratio (FMR)	1.6 (0.5–2.1)	2.6 (2.1–4.8)	<0.001
Visceral-to-subcutaneous fat ratio (VSR)	0.3 (0.1–1.3)	0.6 (0.2–2.9)	0.001
Skeletal muscle mass-to-visceral fat ratio (SVR)	2.5 (1.0–14.2)	1.1 (0.5–2.6)	<0.001
Laboratory test at diagnosis ^1^			
Hemoglobin, g/dL	12.1 (8.3–14.6)	12.5 (9.2–14.3)	0.569
WBC count, 10^3^/uL	7.4 (3.5–15.3)	6.9 (4.1–17.0)	0.971
Neutrophil (%)	69.7 (47.0–83.0)	68.4 (49.7–81.2)	0.646
Lymphocyte (%)	21.5 (9.4–37.0)	21.2 (9.7–42.9)	0.893
Monocyte (%)	7.2 (3.7–16.0)	6.5 (4.5–13.5)	0.557
Platelet count, 10^3^/uL	323.5 (159.0–634.0)	3225 (202.0–564.0)	0.383
Albumin, g/dL	3.9 (2.8–5.0)	4.0 (2.4–5.1)	0.521
Calculated inflammatory index ^1^			
Neutrophil-to-lymphocyte ratio (NLR)	3.2 (1.4–8.8)	3.3 (1.2–8.4)	0.884
Monocyte-to-lymphocyte ratio (MLR)	0.3 (0.1–0.8)	0.3 (0.1–0.9)	0.771
Platelet-to-lymphocyte ratio (PLR)	201.0 (77.2–547.0)	211.2 (97.3–682.1)	0.633
Calculated nutritional index			
Prognostic nutritional index (PNI) ^2^			
Mean ± SD	46.7 (34.5–59.1)	48.2 (27.7–64.0)	0.357

Abbreviations: SD, standard deviation; WBC, white blood cell.

**Table 6:** Factors associated with sarcopenia patients’ survival outcomes.

Characteristics	*n*	(A) Progression-Free Survival						(B) Overall Survival					
		Univariate Analysis			Multivariate Analysis			Univariate Analysis			Multivariate Analysis		
		HR	95% CI	*p*	aHR	95% CI	*p*	HR	95% CI	*p*	aHR	95% CI	*p*
Age, years													
<58	44	1	−	−	1	−	−	1	−	−	1	−	−
≥58	32	1.934	1.158–3.229	0.012	1.905	1.065–3.407	0.030	1.593	0.669–3.795	0.293	1.041	0.417–2.598	0.932
FIGO stage													
III	54	1	−	−	1	−	−	1	−	−	1	−	−
IV	22	1.063	0.612–1.845	0.829	0.917	0.484–1.739	0.791	1.394	0.557–3.488	0.487	0.947	0.345–2.594	0.915
CA-125, IU/ml													
<800	39	1	−	−	1	−	−	1	−	−	1	−	−
≥800	37	0.933	0.563–1.546	0.787	0.863	0.492–1.514	0.608	0.999	0.424–2.354	0.998	1.171	0.414–3.314	0.766
Primary treatment strategy													
PDS	63	1	−	−	1	−	−	1	−	−	1	−	−
NAC	13	1.456	0.773–2.742	0.245	1.254	0.594–2.644	0.553	2.933	1.177–7.309	0.021	3.310	1.096–10.000	0.034
Residual tumor after PDS/IDS													
No gross	47	1	−	−	1	−	−	1	−	−	1	−	−
Gross	29	2.274	1.363–3.795	0.002	2.270	1.334–3.861	0.003	3.587	1.442–8.922	0.006	4.377	1.655–11.578	0.003
BMI, kg/m^2^													
Normal (18.5−22.9)	46	1	−	−	1	−	−	1	−	−	1	−	−
Overweight (23.0−24.9)	22	0.921	0.517–1.641	0.780	0.846	0.440–1.624	0.615	1.024	0.383–2.740	0.962	0.783	0.244–2.517	0.682
Obesity (≥25.0)	8	1.407	0.648–3.051	0.388	0.937	0.370–2.376	0.892	1.726	0.482–6.178	0.401	0.356	0.065–1.935	0.232
Fat-to-muscle ratio (FMR)													
<2.1	38	1	−	−	1	−	−	1	−	−	1	−	−
≥2.1	38	1.262	0.762–2.092	0.366	1.073	0.576–1.999	0.825	2.476	0.989–6.199	0.053	3.377	1.170–9.752	0.024

Abbreviations: aHR, adjusted hazard ratio; BMI, body mass index; CA-125, cancer antigen 125; CI, confidence interval; FIGO, International Federation of Gynecology and Obstetrics; HR, hazard ratio; IDS, interval debulking surgery; NAC, neoadjuvant chemotherapy; PDS, primary debulking surgery.
